# Workload and associated factors: a study in maritime port in Brazil**^1^**

**DOI:** 10.1590/1518-8345.1347.2837

**Published:** 2016-11-28

**Authors:** Marta Regina Cezar-Vaz, Clarice Alves Bonow, Marlise Capa Verde de Almeida, Cynthia Fontella Sant'Anna, Leticia Silveira Cardoso

**Affiliations:** 2 PhD, Associate Professor, Escola de Enfermagem, Universidade Federal do Rio Grande, Rio Grande, RS, Brazil.; 3 PhD, Adjunct Professor, Faculdade de Enfermagem, Universidade Federal de Pelotas, Pelotas, RS, Brazil.; 4 PhD, RN, Escola de Enfermagem, Universidade Federal do Rio Grande, Rio Grande, RS, Brazil.; 5 PhD, Adjunct Professor, Universidade Federal do Pampa, Uruguaiana, RS, Brazil.

**Keywords:** Work, Workload, Working Conditions, Working Environment, Occupational Health, Public Health Nursing

## Abstract

**Objective:**

to identify the effect of the mental, physical, temporal, performance, total effort and frustration demands in the overall workload, and in the same way analyze the global burden of port labor and associated factors that contribute most to their decrease or increase.

**Method:**

a cross-sectional, quantitative study, developed with 232 dock workers. For data collection, a structured questionnaire with descriptive, occupational, smoking and illicit drug use variables was applied, as well as variables on the load on the tasks undertaken at work, based on the questionnaire NASA Task Load Index. For data analysis, we used the analysis of the Poisson regression model.

**Results:**

the demands physical demand and total effort showed greater effect on the overall workload, indicating high overall load on port work (134 employees - 58.8%). The following remained associated statistically with high levels of workload: age (p = 0.044), to be employee of the wharfage (p = 0.006), work only at night (p = 0.025), smoking (p = 0.037) and use of illegal drugs (p = 0.029).

**Conclusion:**

the workload in this type of activity was high, and the professional category and work shift the factors that contributed to the increase, while the age proved to be a factor associated with a decrease.

## Introduction

Planning disease and injuries prevention and promotion of healthy conditions and welfare in the area of occupational health, requires metrics that can detect adequate or inadequate working conditions with the potential to trigger illness in specific groups of workers. One of the metrics that assists in the planning of joint actions for the health of these workers is the study of workload, it represents the cost to the individual performing any task^1^. The measurement of that load analysis shows that many times, the process of this set of activities and carrying out certain tasks can cause human costs (fatigue, stress, illness and accidents), ensuring that the task can be performed with high productivity.

Nursing uses this metric to study the work itself, for example, a study conducted in the United States, which assessed the workload of nurses in intensive care units^2^, indicating greater workload for nurses working 12 hours in day shifts, compared to those who worked the same period in night shifts. Still, research in Brazil on the comparison of the work of nurses who performed the nursing process using printed material versus computerized material showed that the computerized way contributed to reducing nurses' workload, being a support system for decision making of clinical practice^3^. 

These studies indicate that nursing produces scientific knowledge about the human costs that subjects suffer to process the practice, considering the focus of the organization of the process and, moreover, the relationship with the technological tools that can increase or decrease the workload. There are gaps in the accumulated knowledge in nursing, as the main object of study is focused on its workforce. The urgency is to produce knowledge, getting scientifically interested, also other production processes, in order to generate knowledge that unfold in daily practice, assistance to adults in relation to the occupational load that can produce injuries and diseases in the worker. 

This is evidenced when investigating the theme in other areas. A study in the textile industry, carried out by Iranian engineers with male workers, shows high workload and also the night shift workers have higher workload than those of the daytime^4^. Research carried out by Belgian sociologists on activities related or not to the school when there is increased teachers' workload, with 45 years or more, showed that the workload of teaching activities is more related to emotional exhaustion than to non-teaching activities^5^. In the health area, in a survey conducted by psychologists in Australia, with railway workers, showed that the high workload contributes significantly to the fatigue of workers^6^. A research also carried out by psychologists, with operators in the control room of the Italian Navy on the workload before and after the adoption of new equipment, it showed that the applied technology helped in improving the performance of operators, despite the amount of work not being the same^6^. 

Some studies also address this work volume separately, by selecting aspects of workload for investigation. In another research, carried out by professionals of occupational health, with elementary and high school teachers from Iran, it was found that the demand for mental demand and frustration at work, workload components, are the largest and are related to emotional problems^7^. In addition, study conducted with longshoremen - dock worker category - run by psychologists, pointed mental and physical workload of these workers, identifying, beyond the physical issue of the work in the port, the mental issue, in relation to the demand of attention and concentration at work^8^. 

The population of dock workers focus of this study is exposed to inadequate working conditions, related to the degree of workload, from the type of activity. Port work involves loading procedures, unloading, transport and storage of goods^9^, such as container movement (garments, meat and computers), liquids (oils and fuels), solids (grain, coal and cement), fractionated products (paper, wood, steel rollers and wind turbines) and *roll on/roll off* (Cars, buses, trucks, agricultural vehicles and cranes)^10^. This activity is responsible for providing products to all over the world, demonstrating its global importance^11^. On the other hand, it presents stressful and dangerous conditions to workers directly involved in this process.

It may be reiterated that the dock workers deal with processes and very specific products, for example fuels. A study conducted in the US showed that benzene exposure occurs mainly in short-term tasks such as the removal of the fuel tank sampling and disconnecting hoses for the handling of goods^12^. The human effort needed to develop numerous tasks involved in port activities, is opposed to the advancement of technological products that are moved by them, i.e. working conditions that still require qualitative and quantitative improvements so that the total effort imposed on the human strength in the port work is minimized. In this perspective, the study of the workload to which dock workers are exposed contributes to the advancement of knowledge of how costly is the port activity for the health conditions of workers. 

Furthermore, this study is relevant because, relative to the workload of the port environment, there is only one study that supports such knowledge in the literature^8^. This study was conducted in Brazil, which shows a gap in scientific knowledge and its circulation in the international context. Furthermore, it addresses longshoremen, only one of among many other categories of dock workers. These characteristics demonstrate the lack of knowledge about this specific population, due to the characteristics of the port work and possible damage to the health of this worker.

Another situation that demonstrates the importance of the study is the possibility to identify workload components that have more effect on the overall workload. In this direction, aimed to identify the effect of the mental, physical, temporal, performance, total effort and frustration demands in the overall workload, just to analyze the global burden of port labor and associated factors that contribute most to their decrease or increase.

## Method

This cross-sectional, quantitative study was developed with dock workers in 2014. The study population consisted of 723 dock workers. For the realization of the sample calculation we subtracted 53 participants that were away from work (without information regarding their return to work in the research period), totaling 579 dock workers. It was considered a random sample with 95% confidence (IC95%), with 232 participants; dock workers who were present on site for job opportunities in the shift (morning, afternoon or night) were included in the study.

This research was made up of foremen, longshoremen and cargo checkers. The work of wharfage involves goods movement activities within the port, longshoremen are responsible for the movement of goods on the decks or in the basements of ships and cargo conferees act both within the vessels and the port facilities, making the verification of the goods^13^. The subjects were interviewed in the workplace, i.e. a seaport area in southern Brazil, in the period from January to November 2014.

For data collection, individual interviews were used, conducted using a structured questionnaire, which presented socio-demographic variables (gender, age, ethnicity - information reported by participants, marital status and education), occupational variables (port occupation, monthly income, working time and shift), dichotomous variables related to the use of tobacco and illicit drugs (without specifying the type used for this study).

These variables, mentioned, were tested in previous research^14^ ^-^ ^15^ and adapted for the current study. To identify the workload in the tasks we applied a validated scale, the *NASA Task Load Index* (NASA-TLX)^1^, which features six demands for measuring workload: mental demand (a task performed has a lot of mental activity, such as decision and calculation), physical demands (the job requires physical activity such as push, pull and control), temporal requirement (how long is required, and even if the pace of work is slow or fast), performance (how successful the employee believes are the activities that the function requires), total effort (mental and physical trouble that the employee has to achieve the level of performance) and frustration (feeling of insecurity, depression, irritation that work can cause). This scale was chosen to measure the workload because it is validated in its conceptual and operational structures, and is one of the most used for this purpose^16^ ^-^ ^17^.

The variables of the structured questionnaire (socio-demographic, occupational, dichotomous on use of tobacco and illicit drugs and workload) were tested and suitable as a set in meetings of the research group and through a pilot study in time prior to data collection, with a sample of ten subjects in different port categories. The main propositions of this preliminary study were designed to assess and enhance the use of the data collection instrument, in regards to its efficacy in the application and cognitive apprehension of the participants its easiness or difficulty in response to the request, just as enhancing qualification of field researchers.

Each demand is composed of 20 steps of 5 points corresponding to values of zero to 100. After referring to a demand point for each workload, the participant makes a pairwise comparison, deciding between 15 pairs of the six possible combinations of demands indicating which is the most important, with respect to the tasks workload. Thus, the points assigned to each of the demands are weighted by the amount of times the participant considered that demand as the biggest contributor to the port workload. Multiplying the load score for each demand by the number of times it has been indicated as important by the worker is a result which, added and divided by 15, indicates the overall workload in percentage^1^. Although the questionnaire for verification is subjective, it is widely used^2^ ^,^ ^4^ ^,^ ^7^ ^,^ ^18^, demonstrating that it is valid and reliable to measure the workload through the overall workload and the different demands that compose it.

To determine high and low levels of port workload, the overall workload values above and below the third tertile (70%) were considered. This strategy was used in another study^19^, in which there was no predetermined cutoff point. However, although there was a cutoff point determined for the load (50%)^1^, it is understood than using above and below the third tertile (70%) facilitates the distinction between participants who have low and high workloads. Thus, it was considered high level in the overall workload, the tasks developed in the port work if the assigned score was higher than 70 on a scale of zero to 100, corresponding to at least 70% of the highest burden.

Data analysis was performed in the IBM program *Statistical Package for Social Science* (SPSS), version 21.0. Quantitative variables were expressed as average and standard deviation (SD) or median and interquartile range and categorical variables are described by absolute and relative frequencies. The test *t* Student for independent samples was used to compare average between groups - workers with high and low levels of workload. In the case of asymmetry, the Mann-Whitney test was performed. When comparing proportions, we used the chi-square test or Fisher's exact. To control confounding factors, we used the Poisson regression analysis.

The criteria for the variable to enter the model was to produce p-value <0.20 in the bivariate analysis and, as to the permanence of the same model, it should provide a p-value <0.10 in the final model. To evaluate the effect of domains on the overall workload we applied the Cohen measurement. The interpretation most used is that if the Standard Size effect (SSE) is less than 0.5 it is considered small, between 0.79 and 0.5 it is considered moderate and equal to or above 0.8 it is great. The level of significance was 5% (p ≤ 0.05).

The study followed the recommendations of Resolution 466/2012 of the National Health Council, which deals with research involving human subjects. The research project was approved (Protocol 23116.004481 / 2013-53) by the Research Ethics Committee, accredited by the National Council of Ethics in Research (CONEP). All study participants were informed of the objectives of and signed in duplicate the Informed Consent Form (IC), guaranteeing their anonymity and privacy of information.

## Results

The study enrolled 232 dock workers. All of them were male. Most of them (n = 130; 47.8%) were white, 54 (19.9%) black, 34 (12.5%) brown, eight (2.9%) yellow-six (2.2%) indigenous. With respect to education, three (1.1%) were illiterate, 67 (24.6%) had incomplete primary education, 35 (12.9%) completed elementary school, 22 (8.1%) completed secondary school, 86 (31.6%) completed high school ten (3.7) completed higher education, and nine (3.3%) completed higher education or more. The mean age was 48.7 years (SD ± 10.4 years). The majority of the sample port included in the job category - wharfage (n = 137; 50.4%); 78 (28.7%) were longshoremen and 17 (6.3%) load conferees. The average working time was 24.2 years (SD ± 11.3 years) and the average financial income was R$ 4,248.76 (SD ± R $ 2,429.27). As for the demands: mental, physical, temporal, performance, total effort and frustration, workload components, all demands showed significant effect on the overall workload (p <0.001). However, by means of standardized size effect, the influence was moderate for frustration level and great for the other demands. The demands with the greatest effect on the global burden were respectively, physical demand and total stress level (Table 1).

**Table 1:** Distribution of workload demands in the tasks undertaken by the dock workers, according to the *NASA Task Load Index* (NASA-TLX). South region, Brazil, 2014

Workload demands	High Levels n (%)	Reduced levels n (%)	High levels of overall worload (n = 138)	Reduced levels of overall workload (n = 94)	P value*	SSE^†^
			Average±sd	Average±sd		
Mental demand	113 (48,7)	119 (51,3)	74,5±22,0	50,3±23,4	<0,001	1,11
Physical demand	138 (59,5)	94 (40,5)	84,2±17,2	52,5±22,7	<0,001	1,71
Time demand	86 (37,1)	146 (62,9)	69,7±24,9	38,8±23,3	<0,001	1,28
Performance	173 (74,6)	59 (25,4)	89,7±14,9	67,7±25,4	<0,001	1,13
Total effort	156 (67,2)	76 (32,8)	87,2±15,0	61,9±62,1	<0,001	1,58
Frustration	77 (33,2)	155 (66,8)	56,6±32,1	36,8±27,9	<0,001	0,60

* Student *t* Test; ^†^ Cohen SSE; sd: Standard deviation; SSE: Standardized Size effect

Related to the overall workload, 134 dock workers (58.8%) reported to undergo high workloads, i.e., score of the workload greater than 70 on a scale of zero to 100, corresponding to, at least 70% of the highest load in percentage terms. The associations of the variables with high and reduced levels of overall workload in the tasks carried out in the docks, are presented in Table 2. There was a significant association of elevated workload of the tasks developed in the port work with the age (p = 0.001), professional category wharfage (p <0.001) and longshoremen (p = 0.042), time of performance in the industry (p = 0.031) and shift (p = 0.048).

**Table 2:**
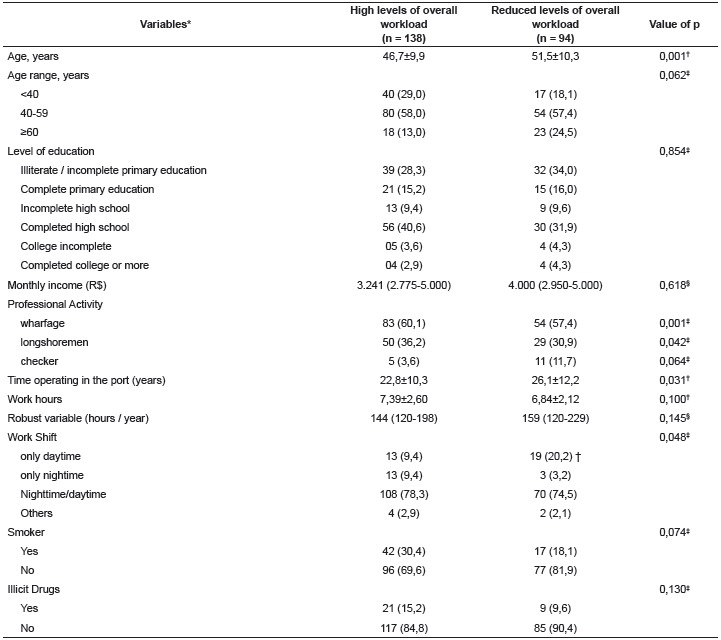
Variables association with high and reduced levels of overall workload in tasks (n = 232). Southern region, Brazil 2014

* Variables described by average ± standard deviation, median (25-75 percentile) or n (%). ^†^ Student t test; ^‡^ chi-square test of Pearson; ^§^ Mann-Whitney test

Younger men with occupation of wharfage and longshoremen with less time of work in the port and that did not only worked the day shift, were more likely to present high levels of workload.

To control confounding factors, the variables with p <0.20 in the bivariate analysis were included in a multivariate Poisson regression. Only variables with p <0.10 remained in the final model. After the adjustment, they remained statistically associated with high levels of workload on the tasks developed in the port work: age (p = 0.044), be a wharfage worker (p = 0.006), work only at night (p = 0.025), smoker (p = 0.037) and use of illicit drugs (p = 0.029) (Table 3).

**Table 3:** Independent factors associated* to high levels of workload on the tasks undertaken by the dock workers. Southern region, Brazil 2014

Variables	PR^†^ (CI^‡^95%)	Value of p
Age, years	0,99 (0,98-1,00)	0,044
Professional Category		
wharfage	1,69 (1,17-2,44)	0,006
Work shift		
Only daytime	1,0	
Only nighttime	1,77 (1,07-2,90)	0,025
Nighttime/daytime	1,26 (0,84-1,88)	0,271
Other	1,35 (0,69-2,61)	0,380
Smoker	1,26 (1,01-1,57)	0,037
Use of illicit drugs	1,30 (1,03-1,66)	0,029

* Poisson regression; ^†^ PR: prevalence ratio; ^‡^ 95% CI: confidence interval of 95%

Dock workers, reduced the prevalence of high levels of workload by 1% (prevalence ratio - PR = 0.99; 95% CI: 0.98 to 1.00) by each additional year. Workers who performed wharfage activities showed an increase in the prevalence of high levels of workload by 69% (PR = 1.69; 95% CI: 1.17 to 2.44), compared to other categories of dock workers.

Those who worked only at night showed prevalence (77%) higher high levels of workload (PR = 1.77; 95% CI: 1.07 to 2.90) compared to those who worked only in the daytime. Also, smoking workers showed prevalence of (26%) higher in the probability of high levels of overall workload when compared to non-smokers (PR = 1.26; 95% CI: 1.01 to 1.57), and those who used illicit drugs showed an increase in the overall workload by 30% (PR = 1.30; 95% CI: 1.03 to 1.66).

## Discussion

The demands: physical demand and total effort, were those that had the greatest effect on the overall workload. The physical demand requirement presents, as a concept the physical request that requires a particular task such as, for example, pushing, pulling and controlling. On the other hand the total effort demand indicates the mental and physical difficulty that the worker faces to reach the level of performance that the activity requires^1^ ^).^ The physical demand is then doubly placed, due to the association of the total physical and mental strain requirements.

In this direction, the physical request of the port work is shown in a study on the constitution of the male working class in labor schemes in private schemes, through the concept of the dock workers as a blue-collar worker, or a worker who performs handwork and requires physical strength to carry out their activities^20^. The physical effort of this worker is shown in a study of the same population, presenting an analysis of secondary data, showing that musculoskeletal disorders are the most frequent^21^. This can present health risks, due to the physical effort made at work.

The mental demand on port work can be explained by the need for workers to be always alert, because, otherwise, there may be negative results, such as accidents, resulting consequences and losses of life. A study conducted in Britain with dock workers showed precarious forms of work, over 35 years, and also the negative consequences of this work for the health and safety of workers^22^. Still, it must be considered that high physical and mental demands, beyond the capacity of individuals, contribute to build a negative work environment^23^. Knowing such working conditions, related to the concept of workload, can help to develop joint strategies between employees and the management of port work, to increase the well-being when carrying out the functions^24^, in order to qualify such conditions in a healthy way. 

Another important result of this study is that the frustration demand was the one who presented a moderate effect on the overall workload. This is because the work on the port can bring satisfaction to workers, i.e. the opposite of frustration (feeling of insecurity, depression, irritation that work can cause). Although not investigated in this study, the question of actual satisfaction may relate no-frustration to satisfaction.

A review of literature on motivation in two professional environments related to sea (sea merchant ships and dock workers), shows that these environments are differentiated from others in terms of motivation. That's because workers can visualize in the port environment, salary rewards and independence, fulfilling factors in relation to the work in the port^25^. 

The results of this study also indicate that the dock worker with one more year in the functions reduced the prevalence of high levels of overall workload by 1%.

Studies on the workload in the textile industry^4^ and teachers^7^ show that age has no significant relevance in the increase / decrease of the overall workload. However, a study in which age was the main factor associated^18^, shows that the worst demand workload is mental, identified by younger workers in a railway company. Because of these results, it defends the idea that there is a need to understand the relationships and workloads at all ages, but it is necessary to pay attention to young people, because of the lack of social support^18^ for this age group.

Another important point in the results, is one in which workers who perform wharfage activities showed an increase in the prevalence of high levels of workload by 69%. The wharfage workers perform tasks involving the movement of goods in facilities within the port, including receiving, internal transport, opening packages for customs inspection, handling, storage, delivery, loading and unloading of vessels^13^. 

What differs from the professional activity of the wharfage from those of other dock workers interviewed (longshoremen and checkers) is that the former perform movement of goods on the decks or in the storage of vessels, i.e. the work of longshoremen occurs just inside of the boats and for workers of the wharfage, work is anywhere in port facilities, except inside the vessels. For the latter, the conferees, the difference happens in the essence of the work, considering that they, as the name says, are responsible for checking the status of the goods, note their characteristics, origin and destination, unlike the wharfage of the workers that should move them^23^. A study focusing on productivity, with terminal operators (longshoremen and carriers) shows that the longshoremen have greater production capacity than the carriers^26^. This last category is similar to wharfage, in relation to work, considering that both act in the port area without entering the vessel. Although the study cited does not identify workloads, it can be assumed that the work performed by operators and wharfage workers impinges them with a high workload, as seen in the increase in the prevalence of workload by 69% in this category of workers, which can contribute to the difficulty in deliveringhigh productivity.

Still, dock workers who work only at night showed 77% higher prevalence of high levels of workload, compared to those working only in the daytime. This result is shown in other studies of the workload with other categories of workers, such as those in the textile industry^4^ and rail workers^6^. This is because shift workers are prone to sleep deprivation, misalignment of circadian rhythms, drowsiness and performance deficits related to sleep, which explains the increase in the overall workload for these workers.

Workers that smoke and workers who used illicit drugs had a prevalence of 26 to 30% higher in increasing overall workload, respectively. This association can occur due to the high overall workload to which dock workers are exposed. Study of different categories (health workers) showed that one of the ways of reducing the use of drugs such as tobacco and psychoactive substances, could be to reduce the workload, demonstrating that the drug issue should be addressed in the workplace^27^. The results of this research instigates further study the specificity the relation between lifestyle, wellness in / work and productivity.

The overall workload of dock workers showed to be high, considering that the majority (58.8%) awarded a grade higher than 70 on the scale of zero to 100. This empirical reality, originated from the concept of global burden of work allowed to introduce a subjective analysis in the objective relation of labor productivity. This characteristic of the workload metrics enables the nature of the dialogue between workers to be addressed with a focus on health without however leaving the stress on productivity.

The workers of this research consider their work directly in relation to productivity and when using language that keeps this nature, it allows him to grasp (subjectivity) that the workload results in organic wear and tear, with potential for disorders and diseases, and as a consequence, reduced productivity^8^ ^,^ ^26^. When doing the interview with the workers and leading them to reflect on the global burden and its consequences, they allowed themselves to this reflect on this approach, leading to promising and ambitious future for academics jointly investing in application of technological intervention with the participation of workers and managers.

It is understood that the study has limitations with respect to the sample, represented by only one seaport, which does not allow generalization. In addition, the cross-sectional study does not verify the causes of the increased workload, but still indicates associations (in this case the professional category wharfage, acting at night, smoking and illicit drug use). It is noted that the theoretical and methodological basis that structured this research is in line with other studies on the workload, thus allowing the nursing profession to dialogue, in an interdisciplinary way with other areas of knowledge. It is expected that the results presented represent a relevant source of information for the development of researches similar to this and other work environments.

## Conclusion

The port workload was found to be high, being the professional category and shift the factors contributing to to its increase, while the age factor was associated with a decrease. Both the characteristics of individuals and the work can influence the port workload. This research alerts for managers and health professionals - among them, nurses - on the high workload to which dock workers are subjected; in their own work language: productivity - and the cost of production, i.e. the workload.
